# Design and Contact Performance Analysis of 3D-Printed Alloy Metal Inertial Micro Switch

**DOI:** 10.3390/mi16050560

**Published:** 2025-05-05

**Authors:** Jinghao Li, Zhipeng Li, Hejuan Chen

**Affiliations:** 1 School of Mechanical Engineering, Nanjing University of Science and Technology, Nangjing 210094, China; leejh1997@njust.edu.cn; 2 School of Mechanical Engineering, Jiangsu Ocean University, Lianyungang 222005, China; 2021000138@jou.edu.cn

**Keywords:** inertial micro switch, deformable electrode, 3D printing, orthogonal experiment, deformation test

## Abstract

In order to reduce space occupation and improve reliability, the modularization and integration of micro switches and their components are a necessary path for development. In this paper, a scheme for an alloy metal inertial micro switch using 3D printing technology is proposed for an integrated design. The switch realizes the turn-on function by causing the deformable electrodes to undergo plastic deformation and make close contact with the outer sleeve under the columnar block extrusion. The influence of electrode structure parameters on electrode contact performance was studied by the orthogonal experimental method. And the best parameter combination scheme for the electrode was determined. The aluminum alloy switch and titanium alloy switch were processed by SLM (selective laser melting) technology. The plastic deformation of the 3D-printed titanium alloy electrode occurred later than that of the 3D-printed aluminum alloy electrode under the same impact. The aluminum alloy electrode underwent plastic deformation and realized stable contact with a response time of 5 µs when the impact load was applied with an amplitude of 627 N and a pulse width of 2.7 ms (simulating high acceleration), which meets the application requirement of the response time being no more than 20 µs. The feasibility of 3D printing technology in high-precision and complex-structure micro switch manufacturing was verified. The research in this paper will provide guidance and reference for engineering applications.

## 1. Introduction

A switch is an electronic component whose primary function is to open and close an electrical circuit so that current can flow or be interrupted. A control switch is a special switch used for systematic process control with a variety of functions, including positioning, self-recovery, and latching. Micro mechanical control switches are becoming increasingly popular and have been widely used in many fields such as household appliances, automotive industry, medical equipment, industrial automation, etc. This type of micro mechanical control switch (referred to as micro switch in this paper) can be classified into various categories according to different standards. This paper focuses on the performance and application of small, ultra-small micro switches.

Micro switch is a kind of snap-action mechanism with tiny contact spacing, short action stroke, low driving control force, and rapid on–off characteristics [1,2,3]. It consists of transmission elements, reeds, contacts, and housings. Transmission elements such as buttons, levers, mass blocks, etc., are used to transmit external mechanical forces to the reed. The reed is the core component of the micro switch and is responsible for converting the external mechanical force into contact action. The contacts are categorized into movable contacts and fixed contacts. The movable contact is fixed on the end of the reed or a protrusion on the end of the reed. And the fixed contact is fixed on the housing or a protrusion on the housing. The housing is used to protect the internal structure of the micro switch against dust, moisture, etc. External mechanical force (such as pressure, tension, inertial force, etc.) is transmitted to one or more pairs of reeds through mass blocks or rods (transmission elements). When the deformation of the reeds after being subjected to a force reaches a certain level (the critical point), it quickly drives the movable contacts to make contact with or be separated from the fixed contacts, thus realizing the connection or disconnection of the circuit.

Micro switches have important applications in the field of high-speed aircraft such as operational unmanned aerial vehicles (UAVs), clustered unmanned aerial vehicles, projectile weapon system, and so on [4,5,6]. The main purpose of these aircraft is to attack enemy targets. The micro switch used in high-speed aircraft is often subjected to a linear inertial force, which is called inertial micro switch. Currently, inertial micro switches are mostly used as control switches for the safety system and the firing control system of a UAV and the weapon system. The inertial micro switches work similarly to the control switches for airbag sensors and igniters in the electronic control of safety airbags in automobiles. According to the different functions, inertial micro switches are divided into two categories: latching type and non-latching type. Latching inertial micro switches have a latching device, which can turn on the circuit when the inertial force exceeds the closing threshold, even if the inertial force disappears later. Non-latching inertial micro switches do not have a latching device, which relies on the two contacts to connect the circuit when in contact and break the circuit when separated. Since there is no precise theory to describe these inertial forces, researchers have mostly used empirical or semi-empirical formulas to describe them, resulting in a significant reduction in the control capability of inertial micro switches due to the large gap between design and practice [7,8].

Scholars have conducted the most research on inertial micro switches manufactured using MEMS technology [9,10]. Reference [11] introduces an earlier MEMS non-latching inertial micro switch under an acceleration below 10*g*. The transmission element is a mass block located in the center with four springs forming four sets of contacts. When the mass block moves up and down, any set of contacts can be closed to connect the circuit. Because the mass block is usually in a suspended state, it will tilt and shake when moving up and down under the action of inertial force, which will seriously affect the stability of contact action and the reliability of contact or separation. Reference [12] utilized a cross-beam structure for contact improvement but could only extend the contact time appropriately and could not improve stability. Reference [13] used an array structure to further improve the contact structure. The array contact structure can appropriately increase the electrostatic adsorption force and extend the contact time. However, it still does not solve the contact stability and contact reliability problems fundamentally. Reference [14] introduces a circular universal inertial switch that uses an equal-line-width gradient beam spring to change the fixed threshold to a variable threshold, which can theoretically improve contact reliability. However, the anisotropic distribution of the threshold in the spatial domain varies greatly, resulting in significant design errors. Reference [15] is a circular universal switch suitable for automotive airbags, with a designed closing acceleration threshold of approximately 40*g*. It uses Archimedean spiral springs and a single circular mass block to design arc-shaped contact-surface fixed contacts and serrated movable contacts. This scheme has good consistency in the radial acceleration threshold and is effective in improving contact stability, but the reliability is reduced due to the large number of contacts. The above MEMS inertial micro switches, all of which are non-latching inertial switches with small thresholds, are not suitable for working conditions with harsh operating environments. The flight acceleration of high-speed aircraft is above 50*g*, even up to tens of thousands to hundreds of thousands of g, and the switch operation is affected by a high impact. The inertial force is also affected by flight conditions and has random variation errors, which have not been successfully solved in theory and manufacturing, hindering its use in high-speed aircraft. Scholars have also proposed other schemes, such as the microfluidic inertial micro switch and magnetic fluid inertial micro switch introduced in references [16,17]. However, these two schemes are still immature in terms of mechanism and manufacturing, and there are a number of key issues that have not been solved.

With the development of science and technology, the main development trend of automatic control switches is intelligent, integrated, wireless, and energy-saving. In order to reduce space occupation and improve reliability, the modularization and integration of micro switches and their components are a necessary path for development [18,19]. With the emergence of additive manufacturing technology, the integrated design of micro switches becomes possible. Therefore, we propose a scheme for an alloy metal inertial micro switch using 3D printing technology. In this paper, Section 2 introduces the structure of the micro switch with integrated deformable electrodes and preliminarily determines the electrode size through deformation force analysis. In Section 3, the orthogonal experimental method is used to analyze the influence degree of electrode parameters and determine the best parameter combination scheme. In Section 4, electrode deformation tests are conducted on the 3D-printed aluminum alloy switch sample and titanium alloy switch sample to obtain the deformation limit impulse of the electrodes. The electrical performance of the aluminum alloy switch is also tested, and the result meets the requirement for rapid response.

## 2. Inertial Micro Switch Design

### 2.1. Inertial Micro Switch with Integrated Electrode Structure

**Figure 1:**
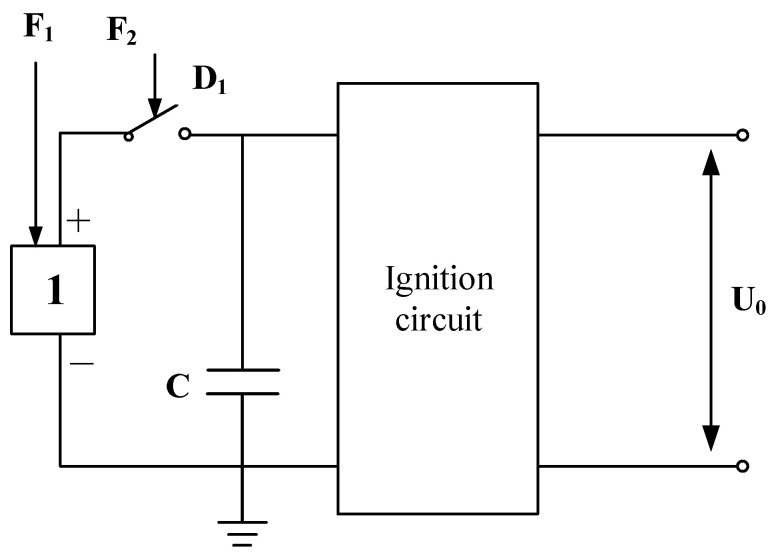
Inertial micro switch control circuit.

There are two technical approaches to utilizing inertial force in inertial micro switches. One is to use inertial force as the working power of the transmission element (mass block), that is, to use inertial force as the energy for the movement of the mass block. Another is to use inertial force as the starting force of the mass block, that is, to use inertial force as the starting information of the mass block, and the energy for the movement of the mass block is provided by springs or other mechanical energy. Whether the first kind of inertial force is implemented as a working force or the second kind of inertial force is implemented as a starting force, there must be a determined environment for inertial force. Therefore, this article introduces an application in the military field, as shown in Figure 1.

Figure 1 shows the ignition control circuit of a high-speed aircraft. The inertial micro switch is used to control the ignition circuit connection to the power. During the launch phase, the environmental force signal $F_{1}$ generated by the acceleration of the aircraft is first sensed by an environmental sensor. $F_{1}$ is used to motivate the dedicated power supply, 1, to work. Another sensor of the aircraft outputs an environmental force signal $F_{2}$ generated by acceleration during the flight phase, which is used to control the inertial micro switch $D_{1}$ to turn on the circuit. To ensure safety during launch, the $F_{1}$ and $F_{2}$ force signals need to be separated.

**Figure 2:**
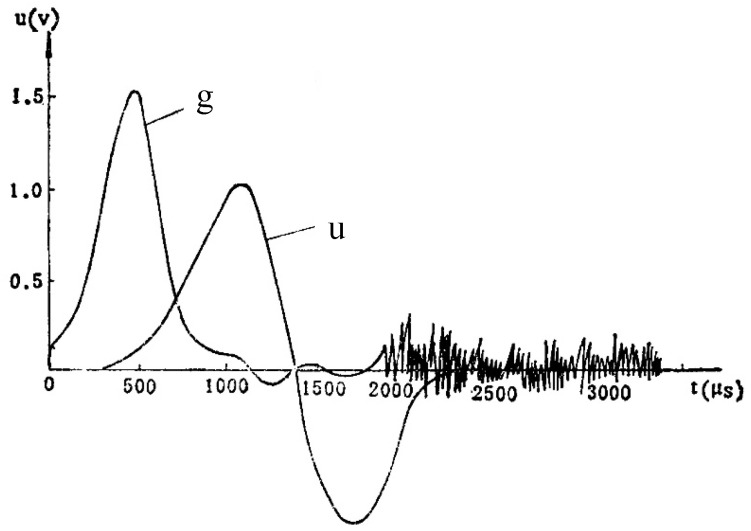
Acceleration curve and dedicated electrical output voltage curve.

Figure 2 shows the acceleration curve of the high-speed aircraft and the output voltage curve of the dedicated power supply [20]. The acceleration of the aircraft is represented by the g-curve. The dedicated power supply is excited to start working before the maximum acceleration point (500 µs). After a delay time, the dedicated power supply outputs an AC voltage signal (u-curve). The voltage value is maximized at about 1000 µs. The turn-on action of $D_{1}$ in Figure 1 is required to be earlier than 500 µs, which ensures the normal operation of the ignition circuit. The dedicated power supply itself does not have the function of energy storage. If the ignition circuit is not connected as soon as possible, a large amount of electrical energy will be lost. Due to the fact that the starting point of the u-curve in Figure 2 is earlier than the highest point of the g-curve, the $F_{2}$ signal controlling $D_{1}$ must be earlier than the starting point of the u-curve. Therefore, the inertial micro switch $D_{1}$ should not only close before 500 µs but also have a very fast response speed. According to the curve in Figure 2, the response time of inertial micro switch $D_{1}$ can be designed to be no more than 20 µs.

**Figure 3:**
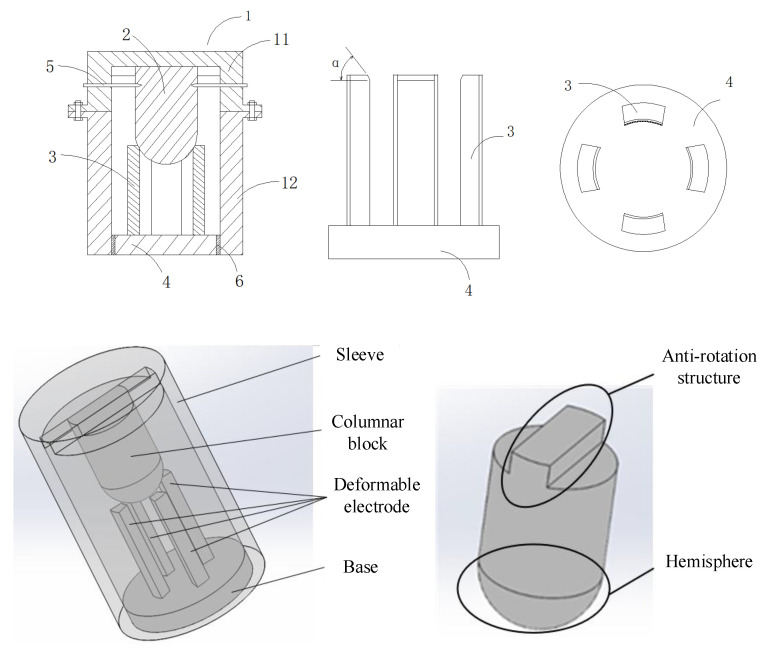
Inertial micro switch.

**Figure 4:**
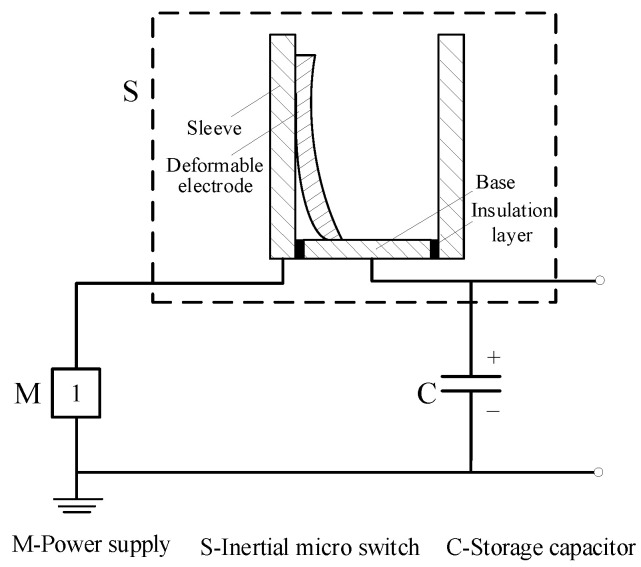
Schematic diagram of switch power connection.

Figure 3 shows the structure of the inertial micro switch. There are two innovations. The first is that the mass block has an anti-rotation function. The second is multiple deformable electrodes with an integrated structure composed of the reed and movable contact. The switch housing (1 in Figure 3) consists of an upper sleeve (11 in Figure 3) and a lower sleeve (12 in Figure 3). Fix and connect the protruding ears on the outer walls of the upper and lower sleeves as a whole through bolts. A columnar block (2 in Figure 3) with an anti-rotation structure and hemispherical structure is the transmission element of the switch. The upper end of this columnar block component is a convex platform. The convex platform cooperates with the groove provided at the upper end of the upper sleeve to prevent the columnar block from rotating inside the sleeve. The lower end of the columnar block is a hemisphere. The columnar block is fixedly connected to the space formed by the upper lower sleeves through the pin (5 in Figure 3) on the side wall of the upper sleeve. Under the hemisphere of the columnar block, the four deformable electrodes (3 in Figure 3) are symmetrically arranged in a circular shape on the base (4 in Figure 3) to ensure uniform force distribution. That is, the deformable electrodes and the base are of a one-piece structure. The four deformable electrodes have exactly the same size. The movable contact is located at the top of the electrode, and the fixed contacts are fixed on the housing. When the electrodes are deformed by the extrusion of the columnar block and touch the sleeve, the micro switch closes. There is an insulation layer (6 in Figure 3) between the base and the lower sleeve. Figure 4 is the schematic diagram of the micro switch power connection. The inertial micro switch relies on the plastic deformation of the electrodes to make contact with the sleeve (the other electrode) to realize the power connection. Theoretically, the hemisphere at the lower end of the column block ensures a uniform force distribution on the electrodes.

The inertial micro switch was produced by additive manufacturing technology (also known as 3D printing technology) in this study. At present, the metal powders used in 3D printing technology are mostly titanium alloy and aluminum alloy. The advantages of titanium alloy in 3D printing are high strength, precise size, and good mechanical properties. The advantages of aluminum alloy in 3D printing include low density, good elasticity, high stiffness and strength, good wear and corrosion resistance, good impact resistance, good electrical conductivity, and excellent formability. The production processes of these two materials are mature. Therefore, this design uses titanium alloy and aluminum alloy for the production of switches.

### 2.2. Deformable Electrode Size Determination

The operation of the inertial micro switch is divided into three stages: the non-contact stage, the initial contact stage, and the deformation stage. The following analysis is the deformation of the electrode under force, focusing on the contact action process in the initial contact stage and the deformation stage.

**Figure 5:**
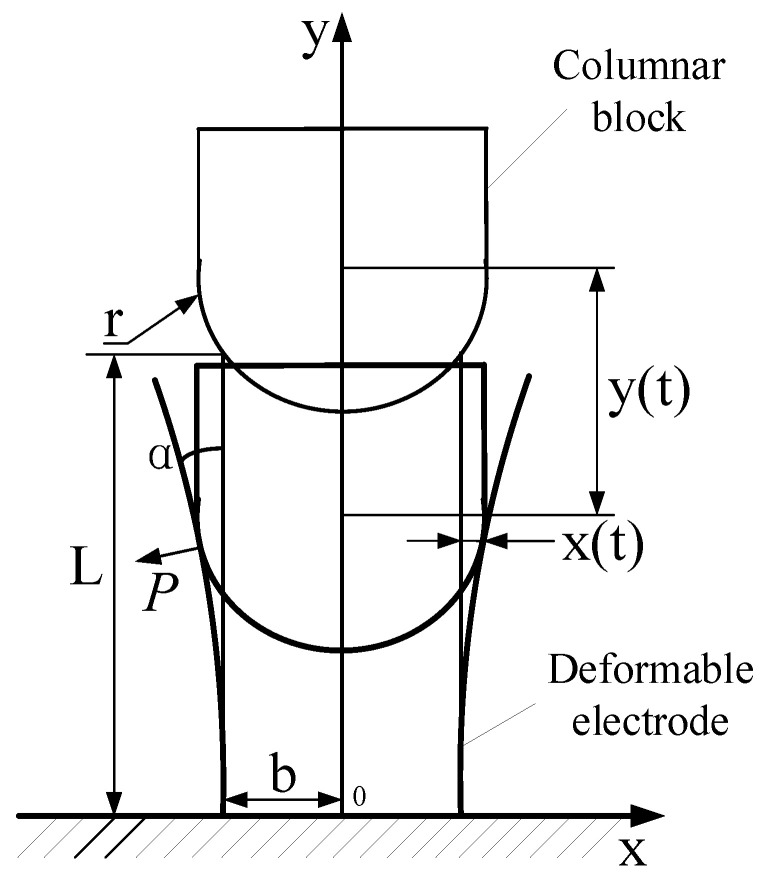
The deformation of the electrode.

The columnar block moves downwards under the inertial force $F_{2}$, causing the pin to break. The hemisphere of the block is in contact with the top of the deformable electrodes, which is the initial contact stage. Define the length direction of the electrode as the y-axis. In order to simplify the model, only two electrodes in relative positions are analyzed, and the direction of outward deformation of the two electrodes is set as the x-axis. Figure 5 is the schematic diagram of the deformation of the electrode. The coordinates of the initial contact point are $\left(b,L\right)$, which is the top of the electrode. At this time, the distance between the center of the hemisphere and the electrode base is *H*. The expression for *H* is

(1) $$H=\sqrt{r^{2}-b^{2}}+L$$

where *b* is the distance between the electrode and the y-axis, *r* is the radius of the hemisphere, and *L* is the length of the electrode.

In the deformation stage, the columnar block continues to move downward to deform the electrode. The columnar block and the electrode are tangential at the contact point. Assume the magnitude of the inertial force acting on the columnar block is *N*. The applied force on the electrode is *P*, and the direction is outward along the normal of the contact point. There also exists a frictional force *f* along the tangent direction of the contact point. The columnar block is subjected to the supporting force $P^{\prime }$ and the frictional force $f^{\prime }$. According to Newton’s third law, we obtain

(2) $$\left\{\begin{array}{c} P=P^{\prime }=\frac{N}{4}\sin\alpha \\ f=f^{\prime }=\mu P \end{array}\right.$$

where μ is the coefficient of friction, and α is the bending angle of the electrode.

Due to the changing inertial force on the columnar block and the electrode bending angle, the magnitude of the force *P* on the electrode is constantly changing. In addition, the moment of inertia of electrodes with the same material but different structural sizes is different. When subjected to the same force *P*, the timing and degree of deformation of electrodes with different structural sizes are different.

Let the coordinates of the contact point at time *t* be $\left(x_{j},y_{j}\right)$. At this time, the center coordinates of the hemisphere of the columnar block are $\left(0,H-y(t)\right)$. y(t) is the displacement of the columnar block along the y-axis. Let x(t) be the deformation displacement of the contact point of the electrode. According to the geometric relationship, the displacement x(t) and y(t) at time *t* can be obtained as

(3) $$\left\{\begin{array}{c} x\left(t\right)=x_{j}-b=r\cos\alpha -b\\ y\left(t\right)=H-r\sin\alpha -y_{j} \end{array}\right.$$

The bottom end of the electrode is fixed to the base and the electrodes bend under force at the contact point, which can be simplified as the cantilever beam model subjected to concentrated stress at the contact point. According to the deflection curve formula, it can be obtained as

(4) $$w\left(y\right)=-\frac{Py_{j}^{2}}{6EI}\left(3y-y_{j}\right)$$

where *E* is the modulus of elasticity of the electrode material, abd *I* is the moment of inertia of the electrode, $I=\frac{WT^{3}}{12}$; $y_{j}\leq y\leq L$.

Therefore, the displacement x(t) of the contact point and the displacement $x_{0}\left(t\right)$ of the top end of the electrode can be obtained as

(5) $$x\left(t\right)=\left|w\left(y_{j}\right)\right|=r\cos\alpha -b=\frac{Py_{j}^{3}}{3EI}$$

(6) $$x_{0}\left(t\right)=\left|w\left(L\right)\right|=\frac{Py_{j}^{2}}{6EI}\left(3L-y_{j}\right)$$

The above is the analysis of the elastic deformation of the electrode. Below is the analysis of plastic deformation. Due to the small size of the switch, the cross-section of the electrode can be approximately rectangular. According to the theory of material mechanics, the critical force *F* exerted on the electrode when the electrode undergoes plastic deformation is obtained as

(7) $$F=\frac{\sigma_{s}WT^{2}}{6y_{j}}$$

where *W* is the width of the electrode, *T* is the thickness of the electrode, and $\sigma_{s}$ is the yield strength of the electrode material.

Taking Equation (7) into Equation (5), the critical displacement $w_{e}$ when the electrode undergoes plastic deformation is obtained as

(8) $$w_{e}=\frac{2\sigma_{s}y_{j}^{2}}{3ET}$$

**Figure 6:**
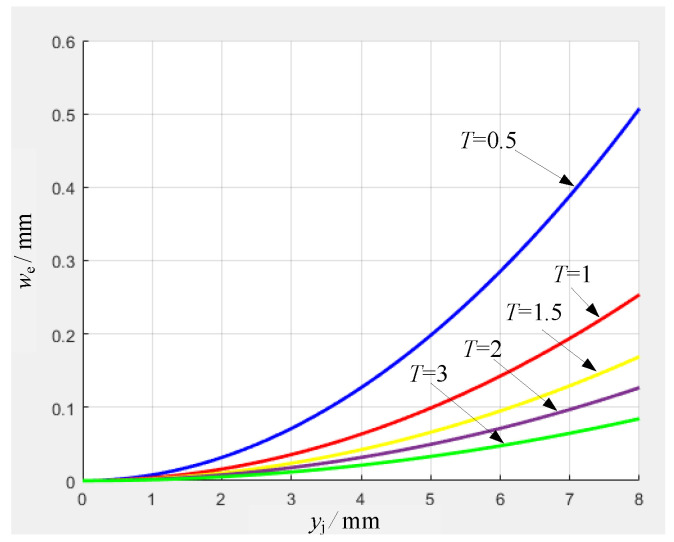
Critical displacement curve with different thicknesses.

According to Equation (8), the effect of different thicknesses shown in Figure 6 is obtained by using the displacement $w_{e}$ as the vertical axis and the coordinate $y_{j}$ of the force point as the horizontal axis. The parameters of the aluminum alloy used are density ρ=2800 ${\mathrm{kg}/m}^{3}$, elastic modulus $E=7.1\times 10^{10}\;$Pa, and yield strength $\sigma_{s}=4.22\times 10^{8}\;$Pa. When the thickness *T* is taken as 0.5 mm, 1 mm, 1.5 mm, 2 mm, and 3 mm, the critical displacement of the top end of the electrode can be obtained in 0.08 mm $<w_{e}<$ 0.51 mm. Therefore, the desired plastic deformation can be obtained by selecting the appropriate electrode thickness.

**Figure 7:**
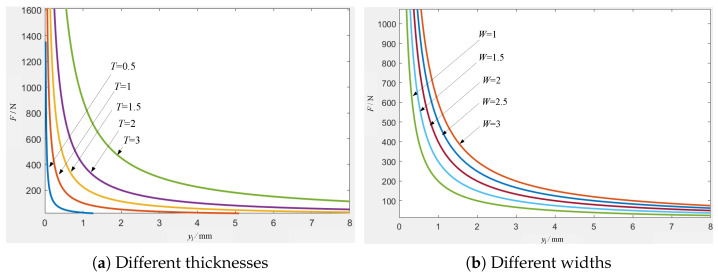
Plastic deformation force curve of electrodes with different thicknesses and widths.

In Equation (7), using the force *F* that causes plastic deformation as the vertical axis and the coordinate $y_{j}$ of the force point as the horizontal axis, the effects of different electrode widths and thicknesses are analyzed. The results are shown in Figure 7.

In Figure 7a, the electrode width *W* is 2 mm, and the thickness *T* is taken as 0.5 mm, 1 mm, 1.5 mm, 2 mm, and 3 mm. The condition for satisfying the plastic deformation of the electrode is $P_{\max}=225\;$N ($y_{j}=4\;$mm, W=2 mm, T=3 mm), $P_{\min}=3.13\;$N ($y_{j}=8\;$mm, W=2 mm, T=0.5 mm). In Figure 7b, the electrode thickness *T* is 2 mm, and the width *W* is taken as 1 mm, 1.5 mm, 2 mm, 2.5 mm, and 3 mm. The condition for satisfying the plastic deformation of the electrode is $P_{\max}=150\;$N ($y_{j}=4\;$mm, W=3 mm, T=2 mm), $P_{\min}=25\;$N ($y_{j}=8$ mm, W=1 mm, T=2 mm).

The results indicate that different thicknesses and widths lead to different plastic deformations of the electrodes. The farther the point of action is from the fixed end, that is, the larger the $y_{j}$ value, the smaller the plastic deformation force required. At 4 mm < $y_{j}$ < 8 mm, the value change rate is small, and the electrode is more prone to plastic deformation at this stage.

**Figure 8:**
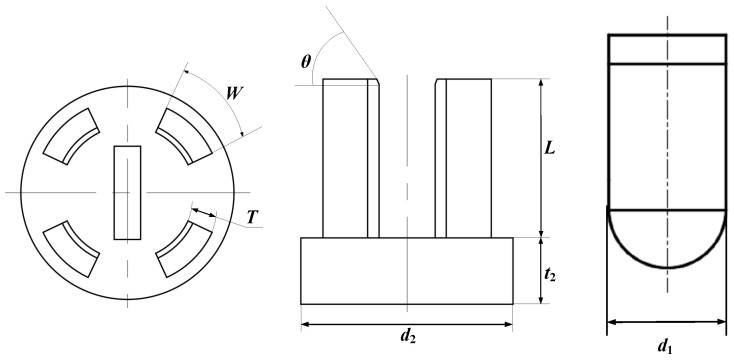
The structural diagram of the inertial micro switch.

The preliminary determination of the size of the switch sample (Figure 8 structure) is as follows: electrode thickness T=0.5∼2 mm, electrode width W=1∼3 mm, hemisphere diameter $d_{1}=3∼6$ mm, electrode length L=8 mm, base diameter $d_{2}=8$ mm, base thickness $t_{2}=2$ mm, pressure angle $\theta =\;45^{\circ }$.

## 3. Analysis of the Influence Degree of Electrode Parameters

### 3.1. Orthogonal Experimental Simulation Parameters

The electrode contact performance greatly affects the electrical performance of the switch. In order to investigate the influence of electrode structure parameters on electrode contact performance, the orthogonal experimental method is used to analyze the influence degree of each parameter. Then, we determine the best combination of structural parameters for the electrode.

The electrode width *W* is taken as 1 mm, 2 mm, 3 mm; the electrode thickness *T* is taken as 0.5 mm, 1 mm, 1.5 mm; and the diameter of the hemisphere $d_{1}$ is taken as 4 mm, 5 mm, 6 mm.

The experimental factors and level arrangements are shown in Table 1.

Design the experiment according to the $L_{9}\left(3^{4}\right)$ orthogonal table. In this orthogonal experimental design, the fourth factor is the blank column, also known as the error column. The orthogonal experimental design table is shown in Table 2.

### 3.2. Best Parameter Combination Scheme

In order to reduce the calculation time of finite element simulation, the columnar block is treated as a rigid body and divided into tetrahedral meshes. The remaining parts of the switch are divided into hexahedral meshes with a higher calculation accuracy. The aluminum alloy electrodes were used in the simulation. The contact area between the electrode and the outer sleeve was used as the assessment result of the orthogonal experimental simulation.

According to the parameter arrangement in Table 1 and Table 2, professional finite element simulation software ABAQUS (version 5.4) was used for the simulation calculations, and the orthogonal experimental simulation results of four factors and three levels were obtained, as shown in Table 3.

In Table 3, $K_{i}$ is the sum of the experimental results corresponding to level *i* in each column. $k_{i}$ is calculated as

(9) $$k_{i}=\frac{K_{i}}{m}$$

where *m* is the number of experiments at level *i* in each column; here m=3.

Let

(10) $$K_{ij}=\left[\begin{array}{c} K_{1}\\ K_{2}\\ K_{3} \end{array}\right],k_{ij}=\left[\begin{array}{c} k_{1}\\ k_{2}\\ k_{3} \end{array}\right],\;i=1,2,3,j=1,2,3,4$$

Range *R* is the maximum minus the minimum of *k* values in each column. The magnitude of *R* value can be used as an indicator to determine the influence of various factors on the experimental results. The larger the *R* value, the greater the impact of this factor on the experimental results. The *R* value of each factor should be greater than that of the blank column. Obviously, the *R* values of factors A, B, and C are all greater than the *R* value of the blank column, indicating that factors A, B, and C are the main factors. And $R_{A}>R_{B}>R_{C}$ means that factor A (hemisphere diameter) has the greatest impact on the experimental results, followed by factor B (electrode width), and the least is factor C (electrode thickness).

Based on the orthogonal experimental simulation results, the parameters of the electrode are further determined through variance analysis. According to the basic principle of variance analysis, we calculate total sum of squared deviations as follows:

(11) $$\left\{\begin{array}{c} T=\sum_{i=1}^{n}x_{i}=\sum_{i=1}^{9}x_{i}=5.9\\ C=\frac{T^{2}}{n}=\frac{5.9^{2}}{9}=3.87\\ \bar{x}=\frac{1}{n}\sum_{i=1}^{9}x_{i}=\frac{5.9}{9}=0.66\\ SS_{T}=\sum_{i=1}^{n}{\left(x_{i}-\bar{x}\right)}^{2}=\sum_{n=1}^{9}x_{i}^{2}-\frac{T^{2}}{n}=3.21 \end{array}\right.$$

The total sum of squared deviations $SS_{T}$ is the sum of the squared deviations of all data from their total mean, reflecting the overall fluctuation of experimental results, which is caused by changes in factor levels and experimental errors.

The column sum of squared deviations is calculated as follows:

(12) $$SS_{j}=\frac{1}{m}\sum_{i=1}^{m}K_{ij}^{2}-\frac{1}{n}{\left(\sum_{i=1}^{n}x_{i}\right)}^{2}=Q_{j}-C$$

$SS_{j}$ reflects the fluctuation of experimental results caused by changes in the level of factors in this column. If the column is a blank column, then $SS_{j}$ represents the fluctuations caused by experimental errors and unobserved factors. In the variance analysis of the orthogonal experiment, the sum of squared deviations of the blank column is usually regarded as the sum of squared deviations of experimental errors, and it is used for significance testing.

The total degree of freedom is $df_{T}=8$. The degree of freedom for the factor in column *j* is $df_{j}=2$.

Calculate variance based on the definition of variance, construct F-statistic, create variance analysis table, and perform significance test. The results are shown in Table 4.

From the results of the variance analysis, it can be seen that factor A (hemisphere diameter) has a highly significant impact, factor B (electrode width) has a significant impact, and factor C (electrode thickness) has no significant impact. The results of this analysis are the same as those in the range analysis.

**Figure 9:**
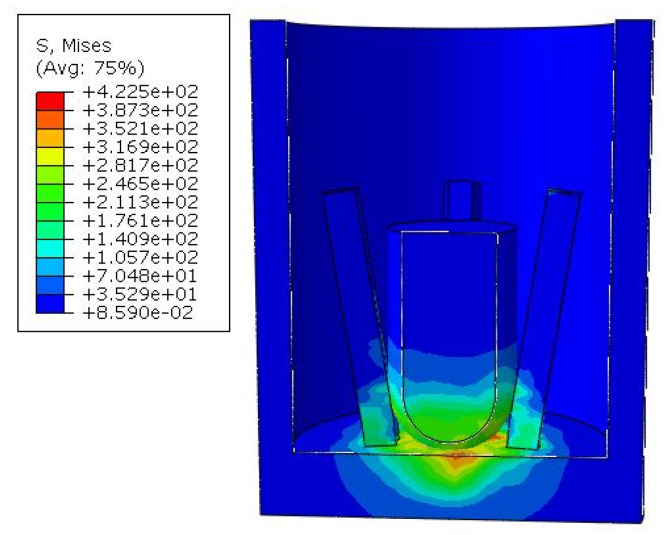
Simulation diagram of non-contact deformation.

There are two main types that greatly affect the closing performance of the electrode. One type is completely non-contact, and the main reason for this result is that the diameter of the hemisphere is too small to fully extend the electrode. The simulation result is shown in Figure 9.

**Figure 10:**
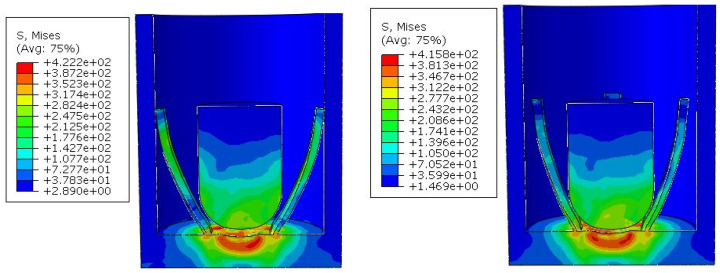
Simulation diagram of unstable contact deformation.

Another type is caused by the large diameter of the hemisphere. When the columnar block reaches the bottom, it squeezes the root of the electrode, causing the top to warp. During the entire force process, the electrode and the outer sleeve make intermittent contact, causing unstable power connection, which seriously affects the performance of the switch. Figure 10 shows the simulation results of the $A_{2}B_{3}C_{1}$ scheme.

Simulation results indicate that in the case of the same width-to-thickness ratio of the electrode, the larger the hemisphere diameter, the larger the contact area. In the case of a larger width-to-thickness ratio, although the outer surface area of the electrode increases, it is very easy to cause the electrode to warp, causing severe shaking in the deformation process and affecting the stability of the switch. Therefore, it is not advisable to choose a larger width-to-thickness ratio in the switch design process, and the hemisphere diameter can be chosen to be larger within the allowable range.

**Figure 11:**
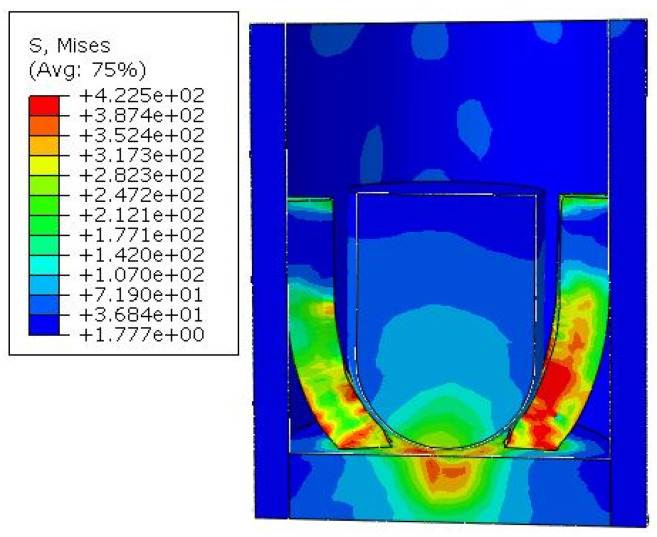
Simulation diagram of electrode deformation under the best parameters.

According to the $k_{i}$ values of various factors in the orthogonal experimental simulation results, the diameter of the hemisphere is selected as level 2, the electrode width is selected as level 3, and the electrode thickness is selected as level 2, that is, the $A_{2}B_{3}C_{2}$ scheme. It is considered that this scheme is the best parameter combination scheme. The simulation result is shown in Figure 11.

**Figure 12:**
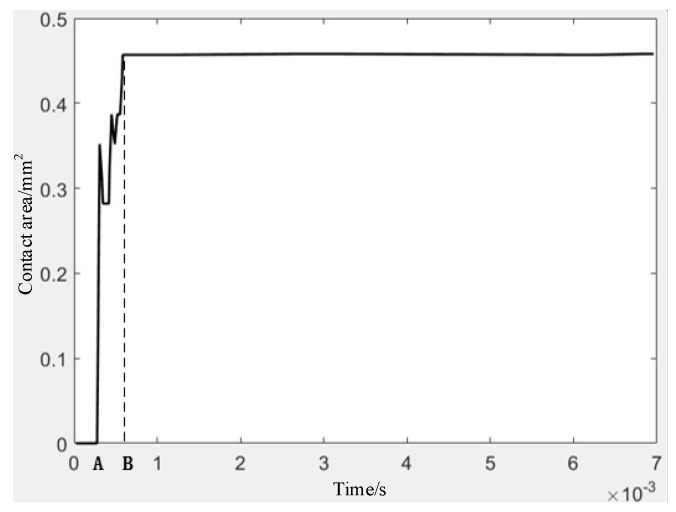
Stable contact curve of electrode.

From the simulation result, it can be seen that the electrode has undergone plastic deformation and is tightly in contact with the outer sleeve. The contact curve is shown in Figure 12. After a very short period of shaking, the contact between the electrode and the outer sleeve is stable, and the contact area is larger than that of nine simulations in the orthogonal experiment. The total contact area of the four electrodes is 1.8 ${\mathrm{mm}}^{2}$.

In summary, the hemisphere diameter, electrode width, and electrode thickness all have an impact on the electrode contact performance. Among them, the hemisphere diameter has the greatest impact, followed by the electrode width, and the least is the electrode thickness.

The best parameter combination scheme for the electrode is the $A_{2}B_{3}C_{2}$ scheme, that is, the hemisphere diameter $d_{1}=5$ mm, the electrode width W=3 mm, and the electrode thickness T=1 mm.

## 4. Electrode Deformation Test and Switch Electrical Performance Test

**Figure 13:**
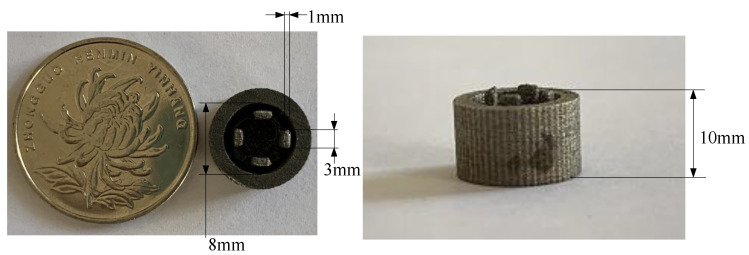
Inertial micro switch sample machined by 3D printing.

The inertial micro switch sample is processed using SLM (selective laser melting) printing technology, as shown in Figure 13. The 3D printing parameters optimized after orthogonal experiments (optimization process detailed in Appendix A) are as follows: the material is aluminum alloy or titanium alloy, laser power is 90 W, scanning speed is 800 mm/s, scanning spacing is 75 µm, layer thickness is 30 µm, and interlayer angle is $67^{\circ }$. The yield strength of the aluminum alloy is $\sigma_{s}=4.22\times 10^{8}$ Pa, and the yield strength of the titanium alloy is $\sigma_{s}=1\times 10^{9}$ Pa.

The exact size of the switch sample (Figure 8 structure) is as follows: electrode thickness T=1 mm, electrode width W=3 mm, hemisphere diameter $d_{1}=5$ mm, electrode length L=8 mm, base diameter $d_{2}=8$ mm, base thickness $t_{2}=2$ mm, pressure angle $\theta =\;45^{\circ }$. The outer ring is a sleeve for an insulated connection to the base. The distance from the top of the electrode to the bottom of the base is 10 mm. The distance from the inner surface of the electrode to the central axis is 1.75 mm.

### 4.1. Electrode Deformation Test Under Simulated Impact

**Figure 14:**
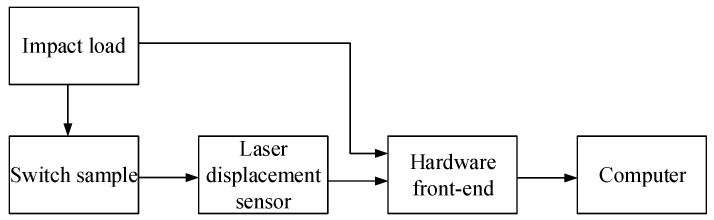
Electrode deformation test principle diagram and testing system.

The principle diagram and testing system of the electrode deformation test are shown in Figure 14. The impact load is generated by the modal force hammer, and the magnitude of each impact force is recorded in the computer. The hardware front-end adopts the M+P VibPilot data acquisition system. The top displacement of the electrode is measured using a laser displacement sensor, and the inertial micro switch is fixed on the test bench with glue. Since the main purpose is to observe the deformation of the electrode, the test is conducted with the steel ball of equal diameter instead of the columnar block. The mass of the steel ball is 5 g and the diameter is 5 mm.

#### 4.1.1. Aluminum Alloy Electrode Ddeformation Test

**Figure 15:**
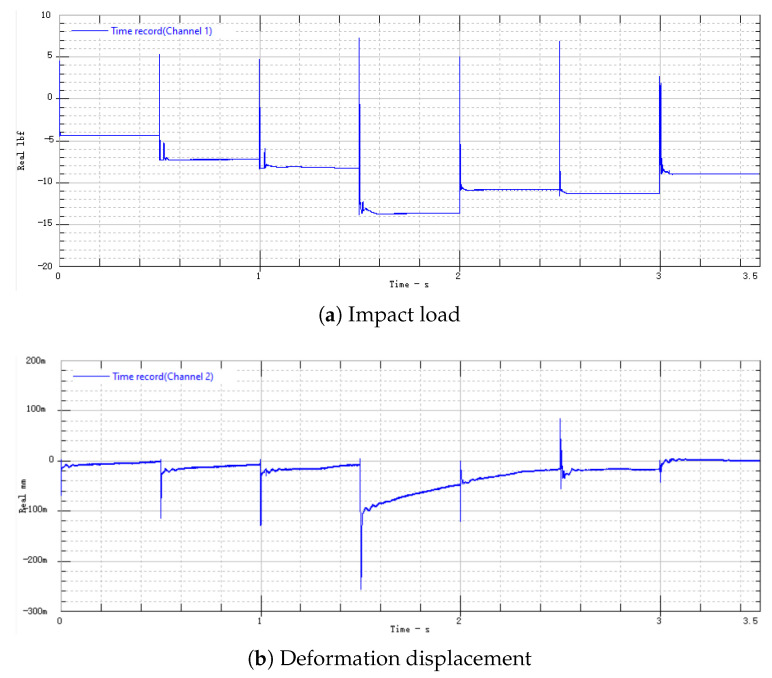
Three-dimensional-printed aluminum alloy electrode impact load and deformation displacement.

Connect the aluminum alloy switch to the testing system shown in Figure 14 and conduct testing. The impact load is shown in Figure 15a, and the electrode deformation displacement is shown in Figure 15b.

**Figure 16:**
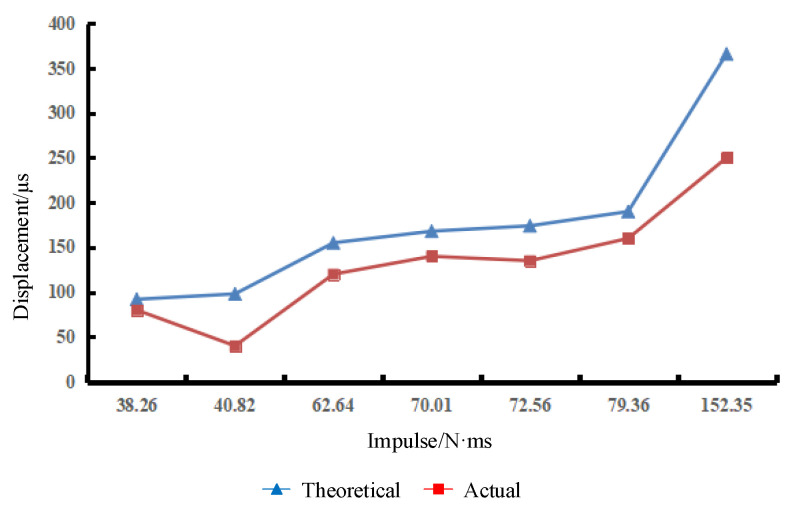
Aluminum alloy electrode impulse–displacement curve.

In the experiment, the aluminum alloy switch is impacted seven times, and the amplitude and pulse width of each impact are recorded in Table 5. Each impact load is fitted as a half-sine function to obtain the fitting impulse. Figure 16 shows the impulse–displacement curve of the aluminum alloy electrode.

**Figure 17:**
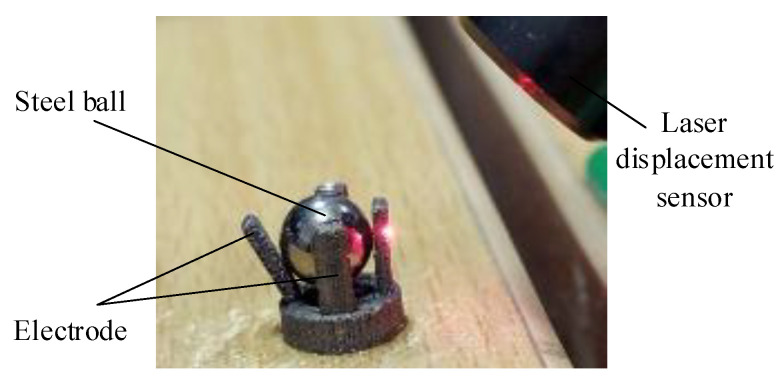
Electrode plastic deformation.

In the seventh impact, with an impact load $F_{p}=152.35$ N·ms, the deformation undergoes a jump. At this time, the electrode undergoes plastic deformation and fracture. Since the maximum range of the laser displacement sensor is 250 µm, the displacement of the electrode actually exceeds 250 µm. The electrode plastic deformation is shown in Figure 17.

In Figure 17, the deformations of the four electrodes are inconsistent. If the mechanical properties of the electrodes are inconsistent, it may lead to the following problems. The difference in response time between different electrodes affects signal synchronization. Local stress concentration leads to premature electrode fracture. The difference in contact resistance causes circuit instability.

The main reason for inconsistent electrode deformation is the influence of manufacturing processes. The analysis is as follows.

(1) Three-dimensional printing has inherent errors. Selective laser melting (SLM) technology suffers from interlayer thermal stress and uneven powder melting, which may result in slight differences in the microstructure (such as grain size and porosity) and macroscopic dimensions (such as width and length) of the electrode. The elastic modulus error range of aluminum alloy electrodes may reach ±5%, while titanium alloy electrodes are more susceptible to thermal stress due to their higher melting point. The connection between the electrode root and the base may experience local stiffness inconsistency due to residual stress. Theoretical analysis and calculations in Section 2.2 also demonstrate that the plastic deformation of an electrode occurs in relation to the electrode’s size and material properties.

(2) The surface roughness of the electrode causes an uneven distribution of the contact force, which can affect the contact resistance and conductivity. If the stiffness of the single-sided electrode is high, the impact force of the columnar block will preferentially act on the low-stiffness electrode, causing the contact pressure distribution to deviate from the theoretical value. Rough surfaces only achieve local contact through asperities, and the actual contact area is much smaller than the apparent area.

**Figure 18:**
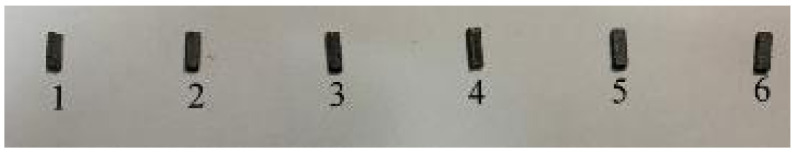
Six electrode samples.

For this reason, six electrode samples were specially made as shown in Figure 18. The test results are shown in Table 6. The surface roughness of the samples was observed and measured using an optical microscope. From the results, it can be seen that there is an error in the size of the electrode samples, with maximum and minimum lengths of 6.06 mm and 6.00 mm, and maximum and minimum widths of 2.17 mm and 2.05 mm. The electric resistances are different, with a maximum value of 0.7652 Ω and a minimum value of 0.7125 Ω.

#### 4.1.2. Titanium Alloy Electrode Deformation Test

**Figure 19:**
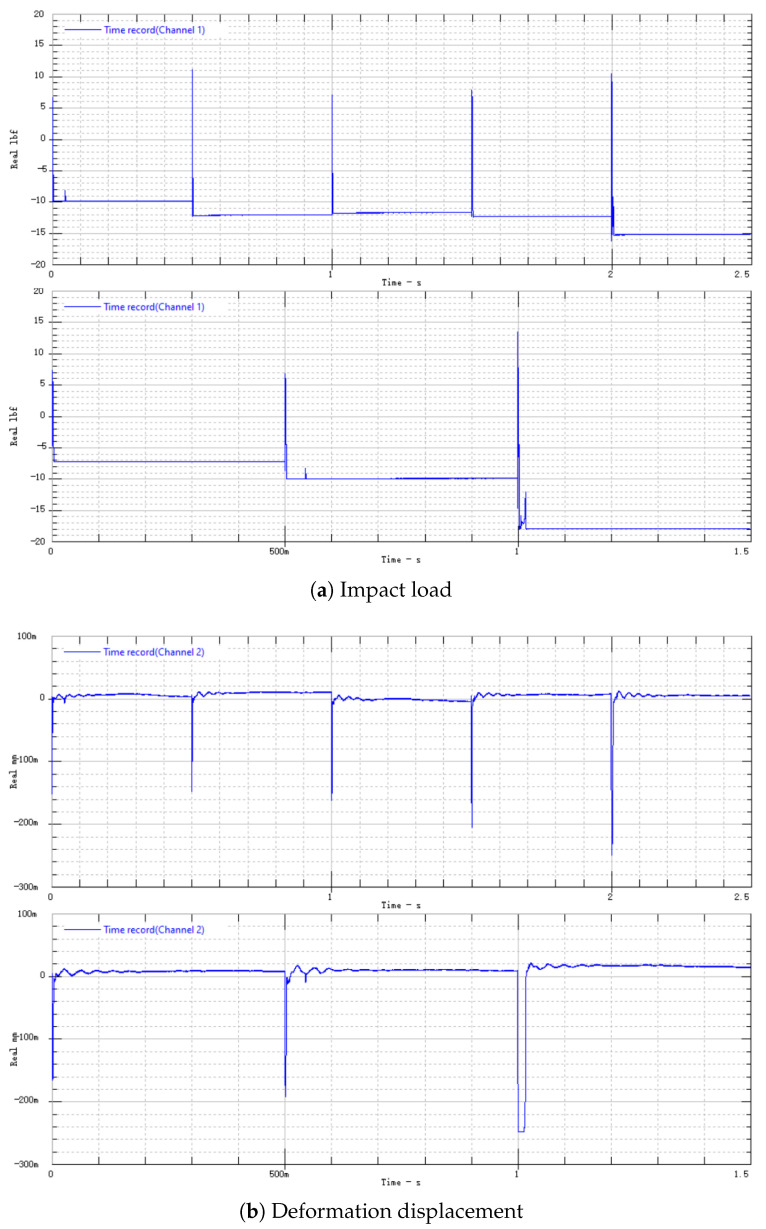
Three-dimensional-printed titanium alloy electrode impact load and deformation displacement.

We used the same testing method for testing the titanium alloy switch. The impact load is shown in Figure 19a, and the corresponding electrode deformation displacement is shown in Figure 19b.

**Figure 20:**
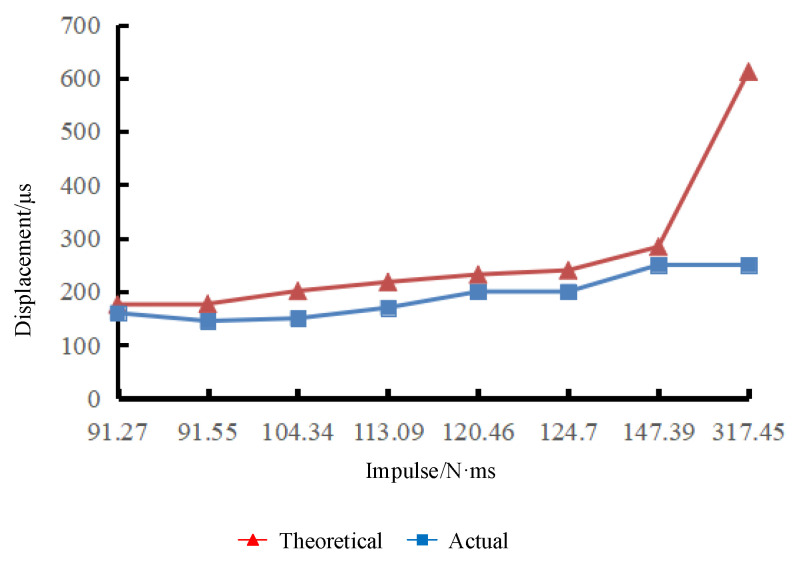
Titanium alloy electrode impulse–displacement curve.

In the experiment, the titanium alloy switch was impacted eight times, and each impact load was fitted as a half-sine function, as shown in Table 7. Figure 20 shows the impulse–displacement curve of the titanium alloy electrode.

In the eighth impact, with an impact load $F_{p}=317.45$ N·ms, the electrode is still within the elastic deformation range, but the maximum displacement of the electrode reaches 250 µm. At this time, the electrode has not undergone plastic deformation and the top of the electrode has not reached the yield stress.

In Figure 16 and Figure 20, the trend of the experimental results curve is consistent with that of the theoretical results curve. There is a deviation between the theoretical and experimental values. The reason for this discrepancy may be that the elastic modulus of the inertial micro switch material is not accurate enough, resulting in a deviation in the theoretical results. In addition, a certain degree of simplification is made in the calculation of impact loads by fitting each impact load as a half-sine function to calculate the impulse value, which is in error from the actual impulse.

In summary, both aluminum alloy and titanium alloy electrodes were made using 3D printing technology. Due to the higher yield strength of titanium alloy compared to aluminum alloy, the plastic deformation time of the 3D-printed titanium alloy electrode under the same impact was later than that of the 3D-printed aluminum alloy electrode.

### 4.2. Switch Electrical Performance Test

**Figure 21:**
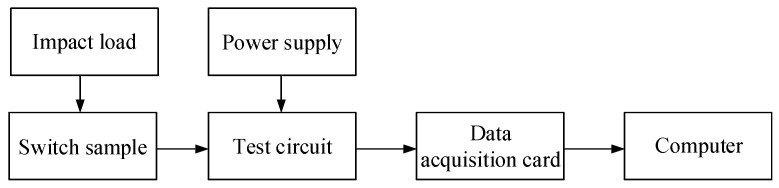
Electrical performance test principle diagram.

The electrical performance test system was built as shown in Figure 21 to verify that the switch can realize a rapid response and stable closure after being subjected to a certain impact load. The aluminum alloy switch was used for testing.

**Figure 22:**
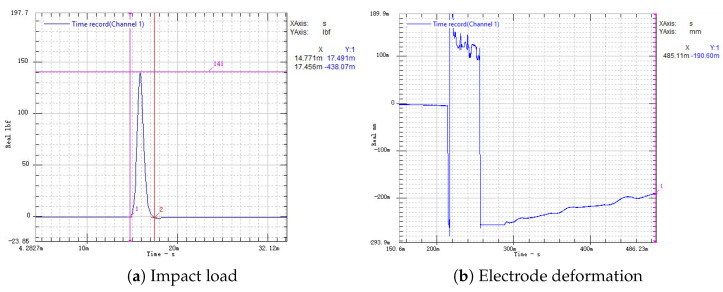
Impact load and electrode deformation curve.

**Figure 23:**
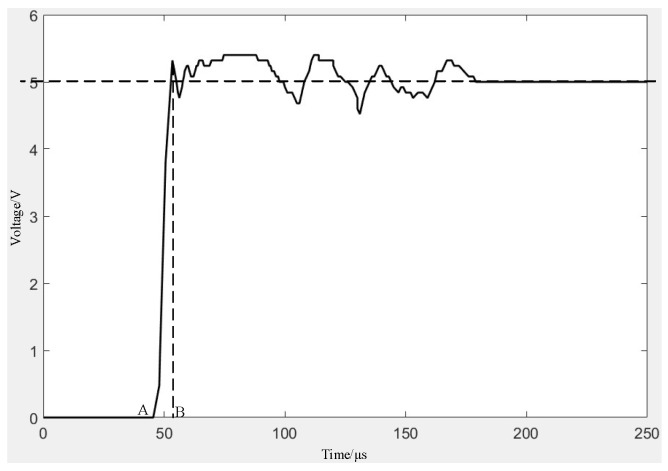
Test circuit voltage variation curve.

The electrical performance test included electrical stability test and response closing time test, with the power supply set to DC voltage 5 V. The M+P company testing system was used to apply the impact load, as shown in Figure 22a, with an amplitude of 627 N and a pulse width of 2.7 ms for simulating the inertial force generated by the high acceleration. The electrode deformation curve is shown in Figure 22b. The electrode did not return to its original state after deformation, indicating that plastic deformation had occurred at this point. The voltage variation curve of the test circuit is shown in Figure 23. The response time from the initial deformation of the electrode (point A) to complete conduction (point B) is about 5 µs, and stable contact is realized after a short period of shaking. The response time of 5 µs meets the application requirements of no more than 20 µs in Figure 1 and Figure 2.

## 5. Conclusions

This article proposes an innovative solution based on 3D printing technology for the design and performance optimization of the inertial micro switch in high-impact environments. This article systematically solves the reliability and response speed of the inertial micro switch in high-impact environments through structural innovation, parameter optimization, theoretical modeling, and impact experiment verification, providing a new technological path for miniaturized control devices in the fields of aerospace military equipment. The specific work and achievements are as follows.

(1) A micro switch structure is proposed, which includes an anti-rotation columnar block and integrated deformable electrodes. The hemispherical contact surface design of the columnar block achieves a uniform force distribution of the electrodes under a high-speed impact, which improves the reliability and stability.

(2) The orthogonal experimental method was used to analyze the influence of electrode parameters on electrode contact performance. The hemisphere diameter of the columnar block and the electrode width and thickness all have an impact on the electrode contact performance. Among them, the hemisphere diameter has the greatest impact, followed by the electrode width and thickness. The best parameter combination scheme for the electrode is the $A_{2}B_{3}C_{2}$ scheme, that is, the hemisphere diameter $d_{1}=5$ mm, the electrode width W=3 mm, and the electrode thickness T=1 mm. The total contact area is 1.8 ${\mathrm{mm}}^{2}$.

(3) Aluminum alloy and titanium alloy switches were processed by SLM (selective laser melting) technology. The plastic deformation impact load limit of the 3D-printed aluminum alloy electrode is $F_{p}=152.35$ N·ms. Under the same impact, the strength of the 3D-printed titanium alloy electrode is higher than that of the 3D-printed aluminum alloy electrode. The plastic deformation of the 3D-printed titanium alloy electrode occurs later than that of the 3D-printed aluminum alloy electrode.

(4) When the impact load was applied with an amplitude of 627 N and a pulse width of 2.7 ms (simulating high acceleration), the aluminum alloy electrode underwent plastic deformation and realized stable contact with a response time of 5 µs. The response time is far below the design threshold of 20 µs, meeting the requirements of highly dynamic scenarios. The feasibility of 3D printing technology in high-precision and complex-structure micro switch manufacturing is verified.

## Figures and Tables

**Table 1:** Levels and values of different structure parameters.

Level	A: Hemisphere Diameter $d_{1}$ (mm)	B: Electrode Width *W* (mm)	C: Electrode Thickness *T* (mm)
1	4	1	0.5
2	5	2	1
3	6	3	1.5
Range	5 ± 1	2 ± 1	1 ± 0.5

**Table 2:** Orthogonal experimental design table.

Header Design	A	B	C	Blank Column
Column number	1	2	3	4
1	1	1	1	1
2	1	2	2	2
3	1	3	3	3
4	2	1	2	3
5	2	2	3	1
6	2	3	1	2
7	3	1	3	2
8	3	2	1	3
9	3	3	2	1

**Table 3:** Orthogonal experimental simulation results of structure parameters.

Header Design	A	B	C	Blank Column	Experimental Results $x_{i}$ (${\mathrm{mm}}^{2}$)
Column number	1	2	3	4	5
1	1	1	1	1	0
2	1	2	2	2	0
3	1	3	3	3	0
4	2	1	2	3	1.05
5	2	2	3	1	0.5
6	2	3	1	2	1.55
7	3	1	3	2	0.63
8	3	2	1	3	0.55
9	3	3	2	1	1.62
$K_{1}$	0	1.68	2.1	2.12	∑=5.9
$K_{2}$	3.1	1.05	2.67	2.18	
$K_{3}$	2.8	3.17	1.13	1.6	
$k_{1}$	0	0.56	0.7	0.71	
$k_{2}$	1.03	0.35	0.89	0.73	
$k_{3}$	0.93	1.06	0.38	0.53	
*R*	1.03	0.71	0.51	0.2	

**Table 4:** Variance analysis of orthogonal experiment simulation results.

Sources of Variation	Sum of Squared Deviations	Freedom Degree	Variance	F-Statistic	Critical Value	Significance
Factor A	1.95	2	0.98	32.67	$F_{0.05}\left(2,2\right)=19.00$	**
Factor B	0.79	2	0.40	13.33	$F_{0.10}\left(2,2\right)=9.00$	*
Factor C	0.40	2	0.20	6.67	$F_{0.10}\left(2,2\right)=9.00$	
Error *e*	0.06	2	0.03			
Sum	3.2	8				

Note: ** means particularly significant. * means significant.

**Table 5:** Aluminum alloy electrode deformation test results.

Serial Number	1	2	3	4	5	6	7
Amplitude (N)	40.05	53.4	57.85	84.55	71.2	62.3	95.68
Pulse width (ms)	1.5	1.2	1.7	1.3	1.6	2.0	2.5
Fitting impulse (N·ms)	38.26	40.82	62.64	70.01	72.56	79.36	152.35
Theoretical value (µm)	92	98	155	168	174	190	366
Experimental displacement (µm)	80	40	120	140	135	160	250

**Table 6:** Electrode samples test results.

Number	1	2	3	4	5	6
Length (mm)	6.02	6.03	6.04	6.00	6.00	6.06
Width (mm)	2.05	2.17	2.14	2.13	2.14	2.10
Thickness (mm)	1	1	1	1	1	1
Electric resistance (Ω)	0.7453	0.7563	0.7346	0.7125	0.7652	0.7514
Surface roughness $S_{a}$ (µm)	16.10	13.22	18.29	16.00	16.97	14.50

Note: These six electrode samples are similar in structure to the electrodes in Figure 3. The reduction in size does not affect the analysis.

**Table 7:** Titanium alloy electrode deformation test results.

Serial Number	1	2	3	4	5	6	7	8
Amplitude (N)	62.3	75.65	102.35	84.55	75.65	89	115.7	142.4
Pulse width (ms)	2.3	1.9	1.6	2.1	2.5	2.2	2.0	3.5
Fitting impulse (N·ms)	91.27	91.55	104.34	113.09	120.46	124.7	147.39	317.45
Theoretical value (µm)	176	177	201.3	218	232	240	284	612
Experimental displacement (µm)	160	145	150	170	200	200	250	250

## Data Availability

All data generated or analyzed during this study are included in this published article.

## Appendix A

The surface roughness of the electrode causes an uneven distribution of the contact force, which can affect the contact resistance and conductivity. The surface roughness is related to the 3D printing parameters. The orthogonal experiment method was used to analyze the effects of laser power, scanning speed, and scanning spacing on surface roughness to determine the optimal combination of printing parameters used in the main text. Due to the repetitiveness of the analysis method and the focus of the paper, this part is included in Appendix A.

The scanning spacing was taken as 0.07 mm, 0.075 mm, 0.085 mm. The scanning speed was taken as 800 mm/s, 1000 mm/s, 1100 mm/s. The laser power was taken as 90 W, 100 W, 120 W. The experimental factors and level arrangements are shown in Table A1.

**Table A1:** Levels and values of different printing parameters.

Level	A: Scanning Spacing (mm)	B: Scanning Speed (mm/s)	C: Laser Power (W)
1	0.07	800	90
2	0.075	1000	100
3	0.085	1100	120

We designed the experiment according to the $L_{9}\left(3^{4}\right)$ orthogonal table. According to the parameter arrangement in the orthogonal table, nine groups of base samples were made, and their surface roughness was measured using an optical microscope. The smaller the surface roughness, the better. The experimental results are shown in Table A2.

In Table A2, the *R* values of factors A, B, and C are all greater than the *R* value of the blank column, indicating that factors A, B, and C are the main factors. And $R_{A}>R_{C}>R_{B}$ means that factor A (scanning spacing) has the greatest impact on the experimental results, followed by factor C (laser power), and the least is factor B (scanning speed).

According to the $k_{i}$ values, the scanning spacing was selected as level 2, the scanning speed was selected as level 1, and the laser power was selected as level 1, that is, the $A_{2}B_{1}C_{1}$ scheme. It is considered that this scheme is the best parameter combination scheme. The scheme is a scanning spacing of 0.075 mm, a scanning speed of 800 mm/s, and a laser power of 90 W. The 3D printing parameters used in the main text are exactly this combination scheme.

**Table A2:** Orthogonal experimental results of printing parameters.

Header Design	A	B	C	Blank Column	Experimental Results $S_{a}$ (µm)
Column number Experiment number	1	2	3	4	5
1	1	1	1	1	33.103
2	1	2	2	2	33.478
3	1	3	3	3	41.965
4	2	1	2	3	16.114
5	2	2	3	1	22.390
6	2	3	1	2	18.286
7	3	1	3	2	20.068
8	3	2	1	3	16.971
9	3	3	2	1	22.536
$K_{1}$	108.546	69.285	68.36	78.029	∑=224.911
$K_{2}$	56.79	72.839	72.128	71.832	
$K_{3}$	59.575	82.787	84.423	75.05	
$k_{1}$	36.18	23.10	22.79	26.01	
$k_{2}$	18.93	24.28	24.04	23.94	
$k_{3}$	19.86	27.60	28.14	25.02	
*R*	17.25	4.5	5.35	2.07	
